# Supplementary material for: Towards a better understanding of the electrosynthesis of 2,5-dicarboxy-2,5-dihydrofurans: structure, mechanism and influence over stereochemistry
Source: R Soc Open Sci. 2019 Jul 10;6(7):190336. doi: 10.1098/rsos.190336 (PMC6689595; doi:10.1098/rsos.190336)
Supplement: Supporting information for Towards a Better Understanding of the Electrosynthesis of 2,5-dicarboxy-2,5-dihydrofurans: Structure, Mechanism and Influence over Stereochemistry; CIFcheck for crystal data [file rsos190336supp1.docx]

**Supporting Information for:**

Towards a Better Understanding of the Electrosynthesis of 2,5-dicarboxy-2,5-dihydrofurans: Structure, Mechanism and Influence over Stereochemistry

Michael A. Shipman, Stephen Sproules, Claire Wilson and Mark D. Symes*

WestCHEM, School of Chemistry, University of Glasgow, University Avenue, Glasgow, G12 8QQ, UK.

*E-mail: mark.symes@glasgow.ac.uk

| *Index* | *Page* |
| --- | --- |
| 1H, 13C and 2D NMR spectra of 2,5-diacetoxy-2,5-dihydrofuran | S3 |
| 1H, 13C and 2D NMR spectra of 2,5-dibutoxy-2,5-dihydrofuran | S8 |
| Figure S10 – cyclic voltammetry | S9 |
| Supplementary Computational Data | S10 |
| Additional single crystal crystallographic data for *cis*-2,5-diacetoxy-2,5-dihydrofuran  spectrum of complex **2** in acetonitrile/THF solution at 150 K | S15 |
| References | S20 |
|  |  |

**Figure S1.** 1H NMR spectrum of *cis*-2,5-diacetoxy-2,5-dihydrofuran in CDCl3.

**Figure S2.** 1H NMR spectrum of a mixture of *trans-* and *cis*-2,5-diacetoxy-2,5-dihydrofuran in CDCl3 obtained *via* chemical synthesis.

**Figure S3.** 13C NMR spectrum of *cis*-2,5-diacetoxy-2,5-dihydrofuran in CDCl3.

**Figure S4.** 13C NMR spectrum of a mixture of *trans-* and *cis*-2,5-diacetoxy-2,5-dihydrofuran in CDCl3 obtained *via* chemical synthesis.

**Figure S5.** HMQC spectrum of *cis*-2,5-diacetoxy-2,5-dihydrofuran in CDCl3.

**Figure S6.** HMQC spectrum of a mixture of *trans-* and *cis*-2,5-diacetoxy-2,5-dihydrofuran in CDCl3 obtained *via* chemical synthesis.

**Figure S7.** COSY spectrum of a mixture of *trans-* and *cis*-2,5-diacetoxy-2,5-dihydrofuran in CDCl3 obtained *via* chemical synthesis.

**Figure S8.** 1H NMR spectrum of a mixture of *trans-* and *cis*-2,5-dibutoxy-2,5-dihydrofuran in CDCl3 obtained *via* electrochemical synthesis.

**Figure S9.** 13C NMR spectrum of a mixture of *trans-* and *cis*-2,5-dibutoxy-2,5-dihydrofuran in CDCl3 obtained *via* electrochemical synthesis.

**
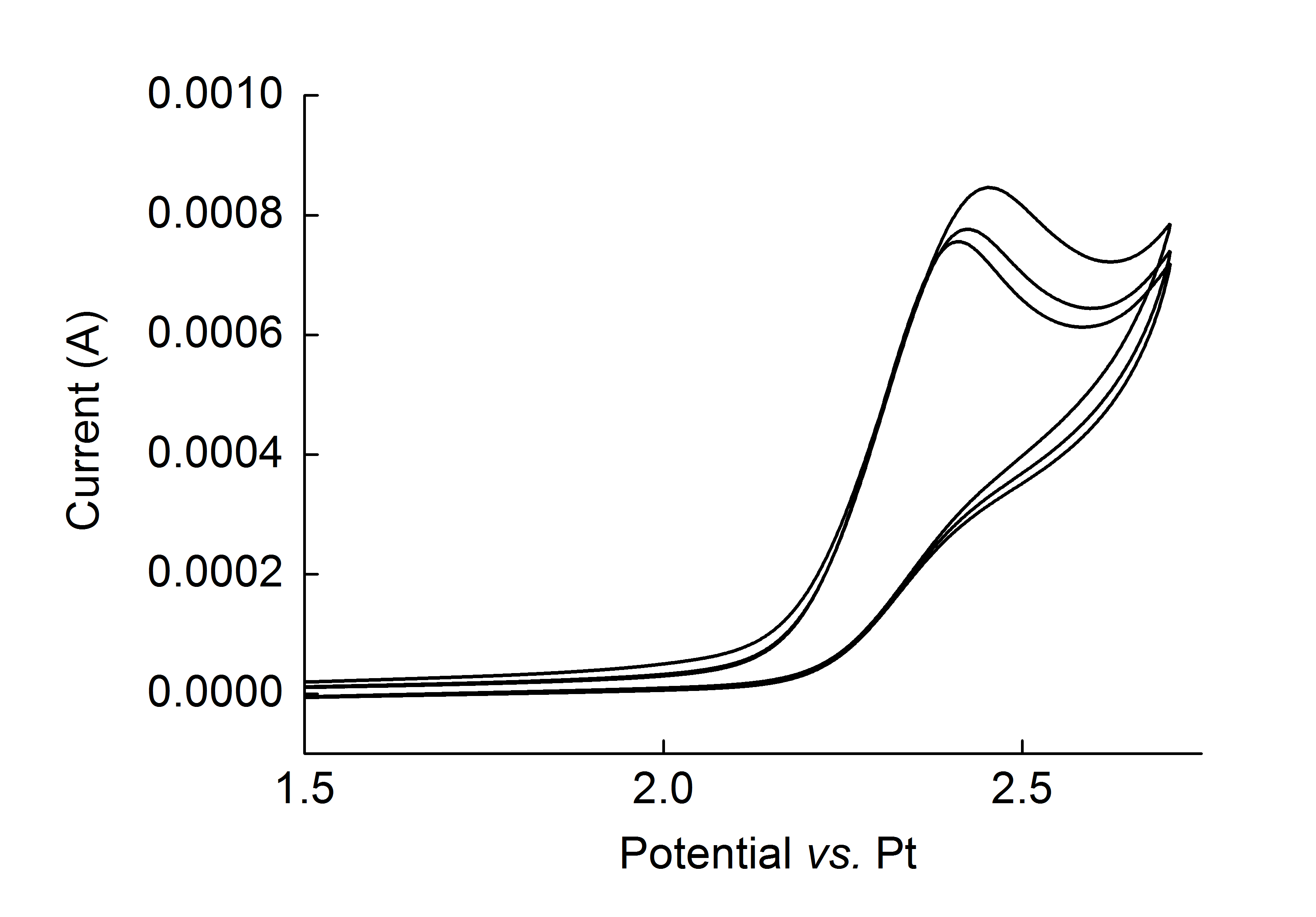
**

**Figure S10.** Cyclic voltammogram of 0.11 M furan in a single chamber cell in a mixed acetate electrolyte (2 g sodium acetate in 10 mL acetonitrile / 40 mL acetic acid) under an atmosphere of nitrogen. The scan rate was 100 mV/s. A Pt wire (area submerged = 1 cm2) was used as the working electrode, with a large surface area carbon counter electrode and a Pt wire reference electrode.

**Supplementary Computational Data**

Geometry Optimized Coordinates for Furan

C -1.80824864713518 1.18328544324190 0.00011953239508

C -0.37469075882240 1.07750643071161 -0.00010138117473

C -0.08714155159193 -0.25699783013190 0.00004363692989

O -1.24815447430304 -0.99513387907364 -0.00001745284793

C -2.28849117222436 -0.09480797473561 -0.00002977173500

H -2.40054186472663 2.09347377423108 0.00019764194279

H 0.34447802827272 1.89125230034324 -0.00021466082149

H 0.83460133246966 -0.82864952790133 0.00015737735687

H -3.28399648193884 -0.52511507568535 -0.00015492204549

Total energy = -229.98146206 Eh

Geometry Optimized Coordinates for Furanium Radical

C -1.37546756220224 1.86058597676796 0.13889118583408

C -0.03714131176468 1.54050828105590 0.28585559904490

C 0.06096371172575 0.14666608950156 0.06889551754110

O -1.16012327287906 -0.38725705425565 -0.20176323860802

C -2.03947702317764 0.64832551949901 -0.16175055487928

H -1.85472164882365 2.83044065241948 0.22911335114645

H 0.79540753431498 2.19692740123955 0.51943193739830

H 0.89930225904897 -0.54552750576724 0.07886844115849

H -3.08116149724242 0.40723972953944 -0.35864919163603

Total energy = - 229.75460866 Eh

Geometry Optimized Coordinates for Furanium Dication

C -1.33914566742290 1.88614291247649 0.14826737183340

C -0.01885027288570 1.56192073901730 0.29192474887618

C 0.07493437616790 0.18102023188911 0.07625234416883

O -1.22207737505797 -0.40048416111189 -0.21513544209537

C -2.04793883885400 0.57680695469328 -0.17517019715449

H -1.85107280907828 2.84510155900215 0.23137912572293

H 0.82601967567981 2.20742060682124 0.52673357764649

H 0.89668595221618 -0.54211329405187 0.07994508893994

H -3.11097385176502 0.38209354126420 -0.36530357093793

Total energy = - 229.38755187 Eh

Cartesian Coordinates for **TS1**

C -1.375467562 1.860585977 0.138891186

C -0.037141312 1.540508281 0.285855599

C 0.060963712 0.146666090 0.068895518

O -1.160123273 -0.387257054 -0.201763239

C -2.039477023 0.648325520 -0.161750555

H -1.854721649 2.830440653 0.229113351

H 0.795407534 2.196927402 0.519431937

H 0.899302259 -0.545527506 0.078868441

H -3.081161498 0.407239730 -0.358649192

O -0.282547581 0.180596687 4.489137538

C 0.412287991 0.034603866 3.436342144

O 0.050838055 0.351148158 2.259348593

C 1.818831422 -0.578341728 3.582481956

H 2.052505676 -0.826611423 4.625858846

H 2.574039494 0.126186924 3.202235282

H 1.894589779 -1.489659884 2.969402387

Total energy = -458.36740651 Eh

Geometry Optimized Coordinates for 2-Acetylfuranyl Radical

C -1.48638333037037 1.58841764178893 -0.12077957774466

C -0.12853358175708 1.42749359117565 0.12182256629350

C 0.10342767444013 -0.00405345718842 0.45770867953988

O -1.18073897538139 -0.64378169402084 0.28685427106476

C -2.09306630405854 0.34303081938490 -0.01200107808121

O 0.44950475087082 -0.16979540934410 1.88277530934526

C 1.61817801213295 -0.75230583571833 2.29314411663903

O 1.78689299955278 -0.84711000791430 3.49850958295502

C 2.61626295304655 -1.23080598736280 1.27149664914237

H -2.00538789870576 2.51311535810562 -0.35890346927573

H 0.65445659515682 2.17987695440035 0.11365381044604

H 0.83607191507893 -0.54741585616931 -0.15011106820177

H -3.12060059000076 0.01584836218334 -0.13452228712748

H 2.20229688112145 -2.06299324584646 0.68311677461839

H 3.50675944514269 -1.58133599664703 1.80204804552251

H 2.89897389273078 -0.43114814782721 0.57365513786410

Total energy = - 458.40312718 Eh

Geometry Optimized Coordinates for 2-Acetylfuranium

C -1.59864633197084 1.43612351333527 -0.16920240533906

C -0.33590203832187 1.67805117649723 0.26236759013765

C 0.32936426408478 0.39202706687443 0.61446330063384

O -0.79009702368525 -0.62442315024354 0.40815947019756

C -1.79898037050747 0.02719911964158 -0.06374233694946

O 0.78248072616115 0.33411739055909 1.91375882469501

C 1.80313039457685 -0.58832547073091 2.29290243403328

O 2.11063885911612 -0.54125003856071 3.45463431840178

C 2.36932855818910 -1.48689871232631 1.24108874552746

H -2.33975287217210 2.14261303474712 -0.52999145281193

H 0.18211070729256 2.63284376706853 0.32981456327194

H 1.06642437029187 0.09799638280963 -0.15102317838163

H -2.67971251283210 -0.55797289049481 -0.33700105845649

H 1.58433050230597 -2.09457154224282 0.76984962559406

H 3.10133512919021 -2.14597890086042 1.71713570166993

H 2.87206207728101 -0.90451365707337 0.45525332077605

Total energy = -458.23565691 Eh

Cartesian Coordinates for **TS2-t**

C -1.592972418 1.435963281 -0.185853997

C -0.330228111 1.677890943 0.245716036

C 0.335038203 0.391866833 0.597811766

O -0.784423090 -0.624583384 0.391507902

C -1.793306452 0.027038887 -0.080393935

O 0.788154704 0.333957156 1.897107304

C 1.808804384 -0.588485706 2.276250943

O 2.116312883 -0.541410273 3.437982837

C 2.375002517 -1.487058948 1.224437271

H -2.334078970 2.142452803 -0.546643066

H 0.187784635 2.632683533 0.313163025

H 1.072098286 0.097836147 -0.167674691

H -2.674038602 -0.558133123 -0.353652683

H 1.590004448 -2.094731778 0.753198127

H 3.107009103 -2.146139137 1.700484249

H 2.877736013 -0.904673894 0.438601861

O -1.068454912 -0.421987390 -2.108438770

C -1.235884475 -0.735793852 -3.327445498

O -0.310424258 -0.975885944 -4.165323953

C -2.681709351 -0.838698502 -3.851293864

H -3.418921816 -0.613720773 -3.069769500

H -2.866484096 -1.851789192 -4.239607878

H -2.824486382 -0.143028087 -4.692578657

Total energy = -686.85446843 Eh

Cartesian Coordinates for **TS2-c**

C -1.592972418 1.435963281 -0.185853997

C -0.330228111 1.677890943 0.245716036

C 0.335038203 0.391866833 0.597811766

O -0.784423090 -0.624583384 0.391507902

C -1.793306452 0.027038887 -0.080393935

O 0.788154704 0.333957156 1.897107304

C 1.808804384 -0.588485706 2.276250943

O 2.116312883 -0.541410273 3.437982837

C 2.375002517 -1.487058948 1.224437271

H -2.334078970 2.142452803 -0.546643066

H 0.187784635 2.632683533 0.313163025

H 1.072098286 0.097836147 -0.167674691

H -2.674038602 -0.558133123 -0.353652683

H 1.590004448 -2.094731778 0.753198127

H 3.107009103 -2.146139137 1.700484249

H 2.877736013 -0.904673894 0.438601861

O -2.502856248 0.047076030 2.001945553

C -3.371225715 0.136519633 2.924129082

O -3.125134195 0.273808893 4.163786430

C -4.857430412 0.075122171 2.520588834

H -4.984332836 -0.047676226 1.437217704

H -5.369219289 0.995278427 2.841011168

H -5.350742721 -0.761841022 3.038506451

Total energy = -686.84963368 Eh

Geometry Optimized Coordinates for *trans*-2,5-diacetoxy-2,5-dihydrofuran

C -1.16188681483011 2.65806055702918 0.19892864367968

C 0.05182559377903 2.31088361174873 0.62487245204403

C 0.22004810117031 0.82573265673963 0.50748459281452

O -1.00190255822555 0.34751159159462 -0.05584491881233

C -1.89235195709133 1.44053536908628 -0.28377561942716

O -2.12153097839456 1.59173821661760 -1.71125148254906

H -1.59963616924252 3.65332550017193 0.16526031364183

H 0.83230191709904 2.95769394115257 1.01952259951963

H 1.06879945816188 0.50646525977400 -0.11267122488642

H -2.85273483840374 1.25132453923867 0.21428057646828

O 0.38359459222412 0.28351624602545 1.84576538321577

C 0.85098649986012 -0.99764467629276 2.02830794761875

O 0.94032612998005 -1.38642332075066 3.17950314068857

C 1.21637357941986 -1.82057183906698 0.82353567545207

C -3.01955522145056 0.77933085279274 -2.36435273258420

C -3.79146634082239 -0.24081512336656 -1.57279276957992

O -3.13513636484826 0.94850493928437 -3.56528771377764

H 0.37430841387465 -1.89115687226279 0.12143763837446

H 1.50164729279713 -2.82128458417193 1.16020325857485

H 2.06313323881584 -1.36685907085769 0.28750801847888

H -4.47235688952017 0.25678174074110 -0.86613273369490

H -3.12138790205308 -0.88753223223116 -0.99095506833081

H -4.38041440429974 -0.84595413899631 -2.26859095692887

Total energy = -686.90202676 Eh

Geometry Optimized Coordinates for *cis*-2,5-diacetoxy-2,5-dihydrofuran

C -1.29787539536271 2.16900305239171 -0.58160221923907

C -0.02424253862330 1.96484979642927 -0.24575811227983

C 0.15351279872392 0.54558664654466 0.20407072056486

O -1.10869404050805 -0.08466387900383 -0.02223678851610

C -2.07578631396932 0.90205925658421 -0.38448640558030

O -3.02802894834537 1.12475419869815 0.69163180327801

H -1.75940549603715 3.09050698697197 -0.92897371462735

H 0.79369627511426 2.68216374184082 -0.25419667597166

H 0.92148248901055 -0.01755309955241 -0.34068226920453

H -2.61241662467693 0.54720830107437 -1.27331818807751

O 0.48977762539329 0.56548462911073 1.61857463924666

C 0.99919429639763 -0.55480127401026 2.23338159085233

O 1.24051441507686 -0.46257567706722 3.42398784908668

C 1.22322585504136 -1.79808324677748 1.41680715631590

C -4.08802466723540 0.26868299722569 0.87837965056345

O -4.82214685439851 0.50643476235507 1.82138196737499

C -4.27902829280533 -0.87863426066729 -0.07562988799449

H 0.30609247228562 -2.10134160072251 0.89446238211558

H 1.54881495911764 -2.59869153340447 2.08759936719740

H 2.00183711525004 -1.62778862439241 0.65833825697609

H -5.11087294302964 -1.49251544287013 0.28328701602399

H -3.37150731428548 -1.49234843182660 -0.15387382097079

H -4.52034773713398 -0.50582323493207 -1.08191811213430

Total energy = -686.90273226 Eh

**Additional single crystal crystallographic data for *cis-*2,5-diacetoxy-2,5-dihydrofuran**


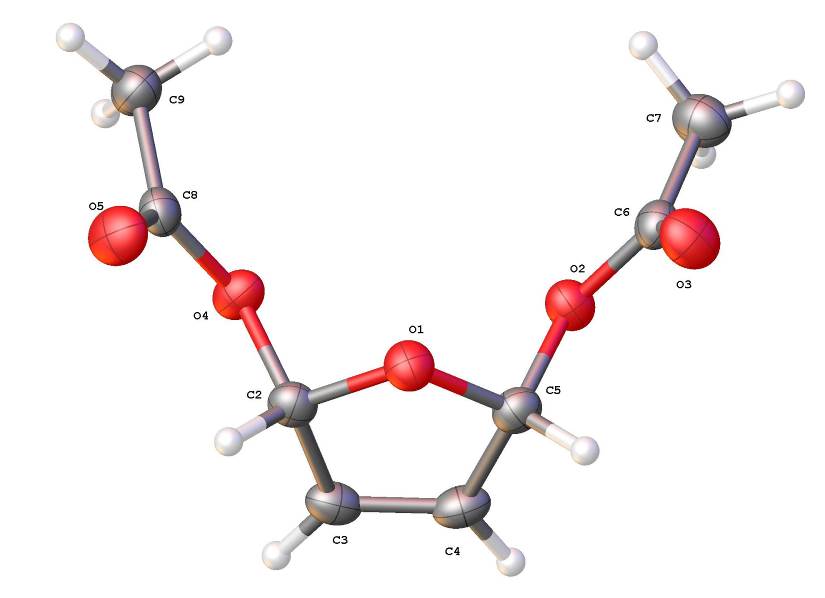


View showing the structure of *cis-*2,5-diacetoxy-2,5-dihydrofuran. Displacement ellipsoids drawn at 50% probability level.

**Refinement**

Crystal data, data collection and structure refinement details are summarized in Table 1.

**Computing details**

Data collection: *APEX3* Ver. 2016.9-0 (Bruker-AXS, 2016); cell refinement: *SAINT* V8.37A (Bruker-AXS, 2016); data reduction: *APEX3* Ver. 2016.9-0 (Bruker-AXS, 2016); program(s) used to solve structure: XT (Sheldrick, 2015); program(s) used to refine structure: XL (Sheldrick, 2015); molecular graphics: Olex2 (Dolomanov *et al.*, 2009); software used to prepare material for publication: Olex2 (Dolomanov *et al.*, 2009).

**References**

Dolomanov, O. V., Bourhis, L. J., Gildea, R. J., Howard, J. A. K. & Puschmann, H. (2009). *J. Appl. Cryst.* **42**, 339–341.S[[1]](#endnote-2)

Sheldrick, G. M. (2015). *Acta Cryst.* C**71**, 3–8.S[[2]](#endnote-3)

Sheldrick, G. M. (2015). *Acta Cryst.* A**71**, 3–8.S[[3]](#endnote-4)

**(2018gu0020_150k_r1)**

*Crystal data*

| C8H10O5 | *F*(000) = 392 |
| --- | --- |
| *Mr* = 186.16 | *D*x = 1.381 Mg m-3 |
| Monoclinic, *P*21/*c* | Mo *K*α radiation,  = 0.71073 Å |
| *a* = 11.7447 (17) Å | Cell parameters from 5942 reflections |
| *b* = 9.7503 (12) Å |  = 2.7–25.3° |
| *c* = 7.8734 (10) Å |  = 0.12 mm-1 |
| β = 96.676 (5)° | *T* = 150 K |
| *V* = 895.5 (2) Å3 | Block, colourless |
| *Z* = 4 | 0.32 × 0.2 × 0.11 mm |

*Data collection*

| Bruker D8 VENTURE  diffractometer | 1631 independent reflections |
| --- | --- |
| Radiation source: microfocus sealed tube, INCOATEC Iμs 3.0 | 1517 reflections with *I* > 2σ(*I*) |
| Multilayer mirror optics monochromator | *R*int = 0.025 |
| Detector resolution: 7.4074 pixels mm-1 | max = 25.3°, min = 2.7° |
|  and  scans | *h* = -1214 |
| Absorption correction: multi-scan  *SADABS2016*/2 (Bruker,2016/2) was used for absorption correction. wR2(int) was 0.1582 before and 0.0643 after correction. The Ratio of minimum to maximum transmission is 0.8875. The /2 correction factor is Not present. | *k* = -1111 |
| *T*min = 0.661, *T*max = 0.745 | *l* = -99 |
| 6125 measured reflections |  |

*Refinement*

| Refinement on *F*2 | Primary atom site location: dual |
| --- | --- |
| Least-squares matrix: full | Hydrogen site location: inferred from neighbouring sites |
| *R*[*F*2 > 2σ(*F*2)] = 0.040 | H-atom parameters constrained |
| *wR*(*F*2) = 0.107 | *w* = 1/[2(*F*o2) + (0.0445*P*)2 + 0.554*P*]  where *P* = (*F*o2 + 2*F*c2)/3 |
| *S* = 1.10 | (Δ/σ)max = 0.001 |
| 1631 reflections | max = 0.36 e Å-3 |
| 120 parameters | min = -0.25 e Å-3 |
| 0 restraints |  |

*Special details*

| *Geometry*. All esds (except the esd in the dihedral angle between two l.s. planes) are estimated using the full covariance matrix. The cell esds are taken into account individually in the estimation of esds in distances, angles and torsion angles; correlations between esds in cell parameters are only used when they are defined by crystal symmetry. An approximate (isotropic) treatment of cell esds is used for estimating esds involving l.s. planes. |
| --- |

*Fractional atomic coordinates and isotropic or equivalent isotropic displacement parameters (Å2)*

|  | *x* | *y* | *z* | *U*iso*/*U*eq |
| --- | --- | --- | --- | --- |
| O1 | 0.27502 (10) | 0.45847 (11) | 0.20054 (14) | 0.0260 (3) |
| O2 | 0.39227 (9) | 0.65113 (11) | 0.25709 (14) | 0.0268 (3) |
| O3 | 0.54271 (11) | 0.51178 (13) | 0.23740 (17) | 0.0367 (3) |
| O4 | 0.14957 (10) | 0.52238 (11) | 0.39486 (14) | 0.0270 (3) |
| O5 | 0.11600 (11) | 0.30235 (12) | 0.45999 (17) | 0.0364 (3) |
| C2 | 0.15928 (14) | 0.48707 (17) | 0.2187 (2) | 0.0249 (4) |
| H2 | 0.108113 | 0.408574 | 0.179267 | 0.030* |
| C3 | 0.13112 (14) | 0.61265 (17) | 0.1134 (2) | 0.0278 (4) |
| H3 | 0.056790 | 0.651233 | 0.088365 | 0.033* |
| C4 | 0.22511 (15) | 0.66125 (17) | 0.0609 (2) | 0.0282 (4) |
| H4 | 0.229359 | 0.740379 | -0.008498 | 0.034* |
| C5 | 0.32422 (14) | 0.57320 (17) | 0.1268 (2) | 0.0255 (4) |
| H5 | 0.370093 | 0.545145 | 0.033274 | 0.031* |
| C6 | 0.50173 (14) | 0.60878 (17) | 0.3021 (2) | 0.0256 (4) |
| C7 | 0.56068 (16) | 0.69836 (19) | 0.4384 (2) | 0.0352 (4) |
| H7A | 0.529621 | 0.791561 | 0.425588 | 0.053* |
| H7B | 0.643053 | 0.700262 | 0.428329 | 0.053* |
| H7C | 0.548128 | 0.662185 | 0.550831 | 0.053* |
| C8 | 0.13694 (13) | 0.41800 (17) | 0.5055 (2) | 0.0252 (4) |
| C9 | 0.15346 (15) | 0.46831 (19) | 0.6857 (2) | 0.0317 (4) |
| H9A | 0.233709 | 0.494960 | 0.715955 | 0.048* |
| H9B | 0.133661 | 0.395190 | 0.762455 | 0.048* |
| H9C | 0.103850 | 0.547820 | 0.696982 | 0.048* |

*Atomic displacement parameters (Å2)*

|  | *U*11 | *U*22 | *U*33 | *U*12 | *U*13 | *U*23 |
| --- | --- | --- | --- | --- | --- | --- |
| O1 | 0.0276 (6) | 0.0195 (6) | 0.0308 (6) | 0.0027 (4) | 0.0032 (5) | 0.0007 (5) |
| O2 | 0.0262 (6) | 0.0251 (6) | 0.0292 (6) | 0.0009 (5) | 0.0033 (5) | -0.0041 (5) |
| O3 | 0.0332 (7) | 0.0334 (7) | 0.0433 (7) | 0.0074 (5) | 0.0034 (6) | -0.0061 (6) |
| O4 | 0.0352 (7) | 0.0214 (6) | 0.0247 (6) | -0.0035 (5) | 0.0048 (5) | -0.0029 (5) |
| O5 | 0.0470 (8) | 0.0230 (6) | 0.0390 (7) | -0.0075 (5) | 0.0043 (6) | -0.0005 (5) |
| C2 | 0.0268 (8) | 0.0240 (8) | 0.0235 (8) | -0.0005 (6) | 0.0011 (6) | -0.0037 (6) |
| C3 | 0.0286 (9) | 0.0293 (9) | 0.0244 (8) | 0.0058 (7) | -0.0025 (6) | -0.0033 (7) |
| C4 | 0.0385 (10) | 0.0247 (8) | 0.0210 (8) | 0.0045 (7) | 0.0013 (7) | 0.0022 (7) |
| C5 | 0.0301 (9) | 0.0242 (8) | 0.0225 (8) | 0.0001 (7) | 0.0044 (6) | -0.0013 (6) |
| C6 | 0.0258 (8) | 0.0245 (8) | 0.0273 (8) | -0.0004 (7) | 0.0068 (7) | 0.0044 (7) |
| C7 | 0.0363 (10) | 0.0316 (9) | 0.0363 (10) | -0.0021 (8) | -0.0019 (7) | -0.0005 (8) |
| C8 | 0.0194 (8) | 0.0264 (9) | 0.0299 (8) | -0.0008 (6) | 0.0033 (6) | 0.0014 (7) |
| C9 | 0.0330 (9) | 0.0347 (10) | 0.0277 (9) | -0.0037 (7) | 0.0050 (7) | 0.0008 (7) |

*Geometric parameters (Å, º) for (2018gu0020_150k_r1)*

| O1—C2 | 1.4110 (19) | C4—H4 | 0.9500 |
| --- | --- | --- | --- |
| O1—C5 | 1.414 (2) | C4—C5 | 1.490 (2) |
| O2—C5 | 1.4413 (19) | C5—H5 | 1.0000 |
| O2—C6 | 1.358 (2) | C6—C7 | 1.490 (2) |
| O3—C6 | 1.201 (2) | C7—H7A | 0.9800 |
| O4—C2 | 1.4463 (19) | C7—H7B | 0.9800 |
| O4—C8 | 1.359 (2) | C7—H7C | 0.9800 |
| O5—C8 | 1.200 (2) | C8—C9 | 1.493 (2) |
| C2—H2 | 1.0000 | C9—H9A | 0.9800 |
| C2—C3 | 1.494 (2) | C9—H9B | 0.9800 |
| C3—H3 | 0.9500 | C9—H9C | 0.9800 |
| C3—C4 | 1.312 (2) |  |  |
|  |  |  |  |
| C2—O1—C5 | 109.25 (12) | C4—C5—H5 | 111.5 |
| C6—O2—C5 | 117.09 (12) | O2—C6—C7 | 110.87 (14) |
| C8—O4—C2 | 117.57 (12) | O3—C6—O2 | 122.87 (15) |
| O1—C2—O4 | 109.18 (12) | O3—C6—C7 | 126.26 (16) |
| O1—C2—H2 | 111.6 | C6—C7—H7A | 109.5 |
| O1—C2—C3 | 105.03 (13) | C6—C7—H7B | 109.5 |
| O4—C2—H2 | 111.6 | C6—C7—H7C | 109.5 |
| O4—C2—C3 | 107.46 (13) | H7A—C7—H7B | 109.5 |
| C3—C2—H2 | 111.6 | H7A—C7—H7C | 109.5 |
| C2—C3—H3 | 125.3 | H7B—C7—H7C | 109.5 |
| C4—C3—C2 | 109.32 (15) | O4—C8—C9 | 110.57 (14) |
| C4—C3—H3 | 125.3 | O5—C8—O4 | 123.13 (15) |
| C3—C4—H4 | 125.2 | O5—C8—C9 | 126.30 (16) |
| C3—C4—C5 | 109.51 (15) | C8—C9—H9A | 109.5 |
| C5—C4—H4 | 125.2 | C8—C9—H9B | 109.5 |
| O1—C5—O2 | 110.14 (12) | C8—C9—H9C | 109.5 |
| O1—C5—C4 | 105.00 (13) | H9A—C9—H9B | 109.5 |
| O1—C5—H5 | 111.5 | H9A—C9—H9C | 109.5 |
| O2—C5—C4 | 106.87 (13) | H9B—C9—H9C | 109.5 |
| O2—C5—H5 | 111.5 |  |  |
|  |  |  |  |
| O1—C2—C3—C4 | -8.05 (18) | C5—O1—C2—O4 | -101.54 (14) |
| O4—C2—C3—C4 | 108.12 (15) | C5—O1—C2—C3 | 13.45 (16) |
| C2—O1—C5—O2 | 101.13 (14) | C5—O2—C6—O3 | 0.7 (2) |
| C2—O1—C5—C4 | -13.59 (16) | C5—O2—C6—C7 | -179.57 (13) |
| C2—O4—C8—O5 | -11.8 (2) | C6—O2—C5—O1 | 84.18 (16) |
| C2—O4—C8—C9 | 167.82 (13) | C6—O2—C5—C4 | -162.30 (13) |
| C2—C3—C4—C5 | -0.26 (18) | C8—O4—C2—O1 | -86.10 (16) |
| C3—C4—C5—O1 | 8.45 (17) | C8—O4—C2—C3 | 160.49 (13) |
| C3—C4—C5—O2 | -108.53 (15) |  |  |

Document origin: *publCIF*.S[[4]](#endnote-5)

1. S O. V. Dolomanov, L. J. Bourhis, R. J. Gildea, J. A. K. Howard and H. Puschmann, *J. Appl. Cryst.* 2009, **42**, 339. [↑](#endnote-ref-2)
2. S G. M. Sheldrick, *Acta Cryst.* 2015, C**71**, 3-8. [↑](#endnote-ref-3)
3. S G. M. Sheldrick, *Acta Cryst.* 2015, A**71**, 3–8. [↑](#endnote-ref-4)
4. S S. P. Westrip, *J. Apply. Cryst.*, 2010, **43**, 920-925. [↑](#endnote-ref-5)
